# Supplementary material for: Genome size versus geographic range size in birds
Source: PeerJ. 2021 Feb 10;9:e10868. doi: 10.7717/peerj.10868 (PMC7881720; doi:10.7717/peerj.10868)
Supplement: Supplemental Information 4 — A file with a phylogenetic tree for all examined bird species (Figure S1), results from partial phylogenetic generalized least squares (Table S1) and results from phylogenetic confirmatory path analysis (Figure S2 and Table S2). [file peerj-09-10868-s004.docx]

**Supplementary material 4**


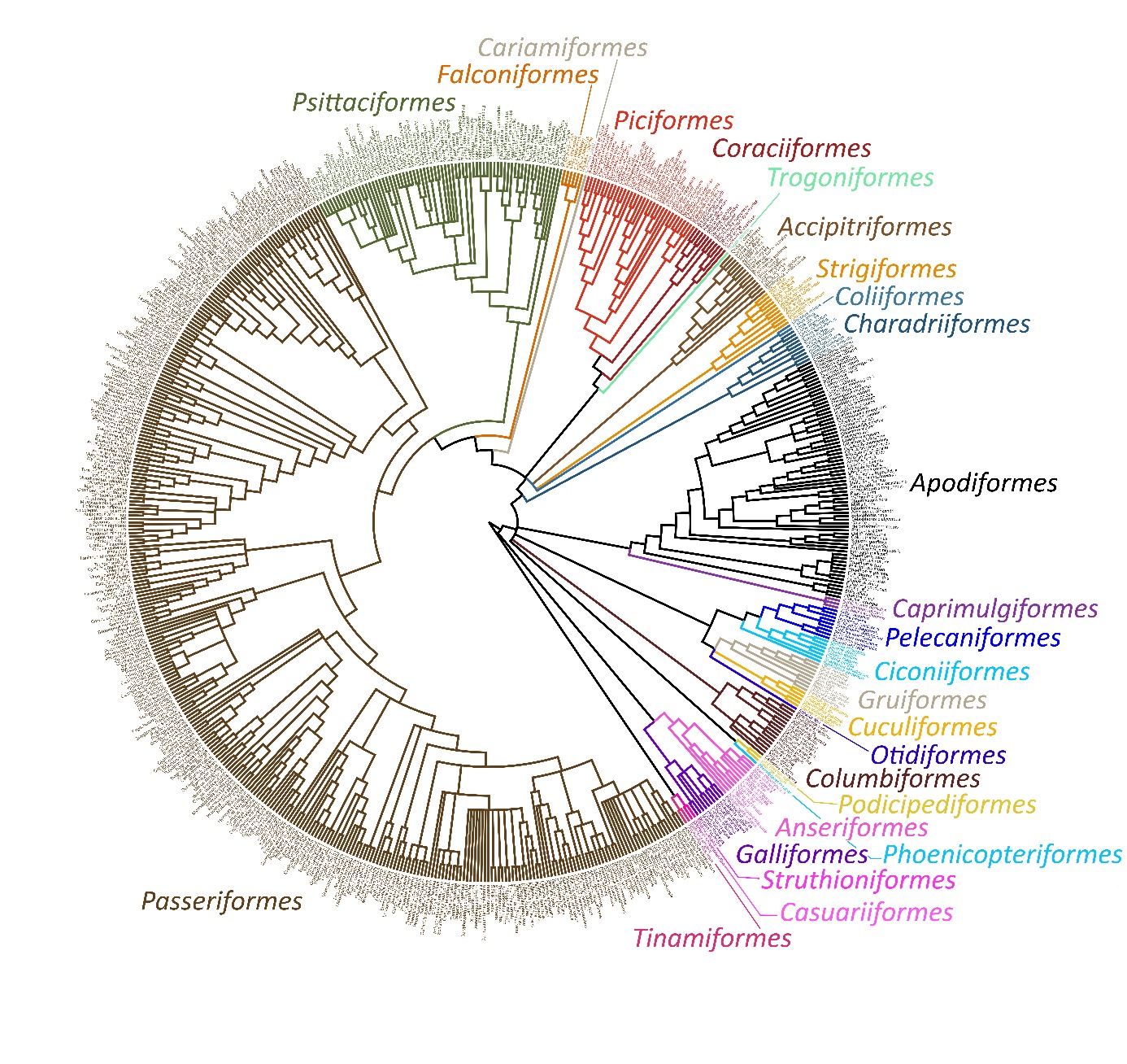


Figure S1. A consensus phylogenetic tree with all examined species.


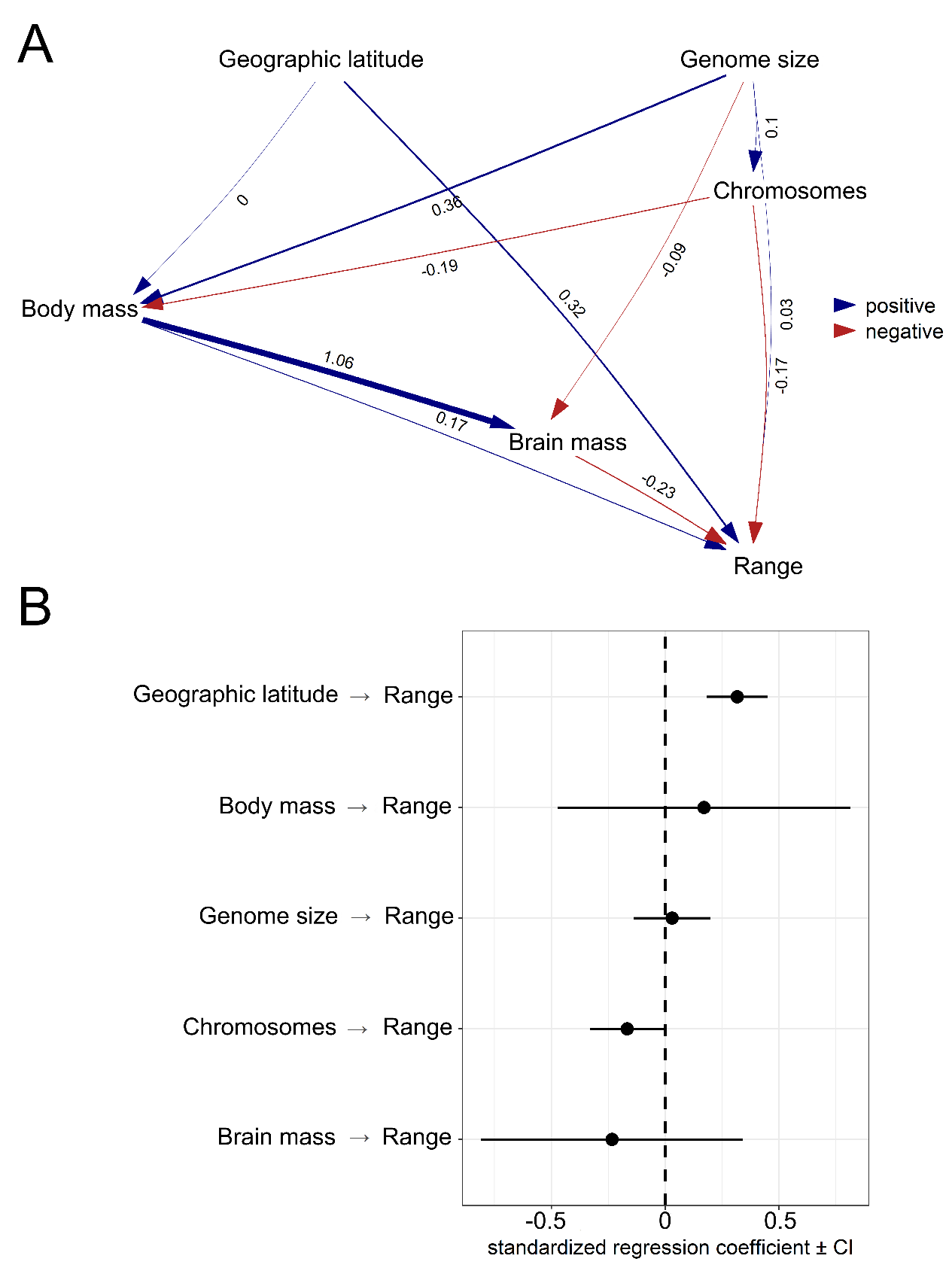


Figure S2. Test supporting causal model with standardized path coefficients (A). Standardized coefficients with 95% confidence intervals (CIs) for explanatory variables associated with geographic range size of birds (B). Analysis based the phylogenetic tree from Ksepka et al. (2020).

Table S1. Phylogenetic generalized least squares models testing association among geographic species ranges in birds and genome size, chromosome number, body mass, brain mass and geographic latitude. Analysis based on the phylogenetic tree from Ksepka et al. (2020).

| **Model 1st (N = 262 species)** |  |  |  |  |
| --- | --- | --- | --- | --- |
| Effect | *estimate* | *SE* | *t* | *P* |
| (Intercept) | -1769163.6 | 18649898.4 | -0.095 | 0.924 |
| Genome size | 18738048.3 | 5077635.0 | 3.690 | **<0.001** |
| Body mass | -1705866.2 | 781220.6 | -2.183 | **0.030** |
| Geographic latitude | 118802.3 | 57736.4 | 2.058 | **0.041** |
| **Model 2nd (N = 254 )** |  |  |  |  |
| Effect | *estimate* | *SE* | *T* | *P* |
| (Intercept) | -6601929.2 | 22323525.6 | -0.296 | 0.768 |
| Genome size | 16141037.3 | 6868117.9 | 2.350 | **0.019** |
| Body mass | -40195.7 | 926822.7 | -0.043 | 0.965 |
| Brain mass (residual) | 9036609.6 | 4282034.0 | 2.110 | **0.036** |
| Geographic latitude | 179524.7 | 64235.0 | 2.794 | **0.006** |
| **Model 3rd (N = 55)** |  |  |  |  |
| Effect | *estimate* | *SE* | *T* | *P* |
| (Intercept) | 27138470.8 | 23192263.6 | 1.170 | 0.248 |
| Genome size | 17644288.8 | 12517961.5 | 1.609 | 0.126 |
| Chromosome number | -397683.4 | 187801.8 | -2.118 | **0.039** |
| Body mass | -1505298.1 | 1469867.9 | -1.024 | 0.311 |
| Brain mass (residual) | -1894156.4 | 6003833.9 | -0.3154 | 0.753 |
| Geographic latitude | 295362.8 | 111703.5 | 2.644 | **0.011** |
| **Model 4rd (N = 55)** |  |  |  |  |
| Effect | *estimate* | *SE* | *T* | *P* |
| (Intercept) | 2769718.2 | 20964743.6 | 0.132 | 0.895 |
| Genome size | 12205449.0 | 12679723.8 | 0.963 | 0.340 |

Table S2. The results of phylogenetic path analysis based on the phylogenetc tree from Ksepka et al. (2020). Different sets of models are compared (see Figure 3) Explanations: *k -* independence claims made by the model, *q* - the number of parameters, *C*- the C statistic, *P* - p-value for *C*, *CICc* - the *C-*statistic information criterion corrected for small sample sizes, Δ*CICc* - the difference in *CICc* with the top model, *l* - the associated relative likelihoods, *w* - *CICc* weights (w). A significant *p* indicates that the available evidence rejects the model.

| Model | *k* | *q* | *C* | *p* | *CICc* | *ΔCICc* | *l* | *w* |
| --- | --- | --- | --- | --- | --- | --- | --- | --- |
| indirect complex | 4 | 17 | 5.658 | 0.685 | 56.659 | 0.000 | 1.000 | 0.897 |
| indirect simple | 6 | 15 | 18.348 | 0.105 | 60.979 | 4.320 | 0.115 | 0.103 |
| null | 15 | 6 | 115.583 | 0.000 | 129.370 | 72.712 | 0.000 | 0.000 |
| direct | 10 | 11 | 105.137 | 0.000 | 133.423 | 76.764 | 0.000 | 0.000 |
